# Selenized neural stem cell exosomes for CNS trauma repair

**DOI:** 10.20517/evcna.2025.156

**Published:** 2026-01-09

**Authors:** Lina Zhu, Kailu Guo, Xi Liu, Yanlin Feng, Cuiping Zhang

**Affiliations:** ^1^Rehabilitation Medicine Center and Institute of Rehabilitation Medicine, West China Hospital, Sichuan University, Chengdu 610041, Sichuan, China.; ^2^Key Laboratory of Rehabilitation Medicine in Sichuan Province, West China Hospital, Sichuan University, Chengdu 610041, Sichuan, China.; ^3^Medical Innovation Research Division, Chinese PLA General Hospital, Beijing 100048, China.; ^4^College of Graduate, Tianjin Medical University, Tianjin 300070, China.; ^5^Key Laboratory of Cellular Physiology at Shanxi Medical University, Ministry of Education, and the Department of Physiology, Shanxi Medical University, Taiyuan 030001, Shanxi, China.; ^#^These authors contributed equally to this work.

**Keywords:** Exosomes, neural stem cells, selenium, traumatic brain injury, spinal cord injury, blood-brain barrier

## Abstract

A recent study on Cell Reports Medicine by Wang *et al*. introduces a hybrid exosome platform - selenized neural stem cell-derived exosomes (SeNExo) - that couples the biological functionality of neural stem cell exosomes with the antioxidant power of ultrasmall nanoselenium. SeNExo crosses the blood-brain barrier via apolipoprotein E (APOE)-lipoprotein receptor-associated protein-1 (LRP1) interaction, scavenges reactive oxygen species, and restores glial-neuron homeostasis. It demonstrates potent therapeutic efficacy in both traumatic brain injury and spinal cord injury mouse models. This work highlights a promising direction for engineering multifunctional, cell-free nanotherapeutics for central nervous system repair.

Neural stem cell therapy has been a leading-edge approach for treating traumatic injuries to the central nervous system (CNS); however, challenges such as poor cell survival and limited differentiation within the pathological microenvironment have restricted its application. By contrast, neural stem cell-derived extracellular vesicles (EVs) - sometimes referred to as “exosomes” (NExo, as termed by Wang *et al*.) - are emerging as a promising alternative^[1,2]^.

According to the Minimum Information for Studies of Extracellular Vesicles (MISEV) 2023 position paper^[3]^, EVs are lipid bilayer-membrane-bound particles derived from cells, capable of transferring small molecules such as lipids, proteins, and nucleic acids. The word “exosome” though widely used, refers to particles originating from the endosomal system and could, though not recommended, refer to a specific type of EVs of certain origin. However, to remain consistent with the original publication by Wang *et al*., we will still use the term “exosomes” in this Research Highlight to avoid confusion^[2]^.

NExo, originating from neural stem cells, is highly structurally stable and could partially recapitulate the therapeutic effects of stem cells. Its capability of crossing the blood-brain barrier (BBB) is another unique advantage for treating traumatic injuries to the CNS. However, natural NExo lacks efficacy and specificity. Taking reactive oxygen species (ROS) as an example, the intrinsic ROS-scavenging capacity of natural exosomes is relatively modest and therefore insufficient for treating traumatic brain injury (TBI). How to enhance the specific ROS-scavenging ability while preserving the advantage of natural NExo is a crucial question.

To address this issue, Wang *et al*. present an elegant strategy that integrates nanotechnology and exosome biology to form a modified NExo^[2]^. Recognizing that nanoselenium can scavenge oxidative stress^[4]^, they engineered selenized NExo (SeNExo) by anchoring ultrasmall nanoselenium directly onto the exosomal membrane via a one-pot approach. The approach involves incubating NExo with sodium selenite, followed by exposure to ascorbic acid. This enables phosphoryl groups on the exosomal membrane to interact with the selenite precursor, leading to *in situ* growth of ultrasmall nanoselenium. It allows growth of ultrasmall nanoselenium (∼3.5 nm) at the membrane while avoiding organic solvents, ensuring the biological activities of the NExo components. This hybridization not only preserves the biological cargo of NExo but also introduces a selenium–oxygen (Se–O) bond that significantly enhances ROS scavenging efficiency - nearly fourfold higher than conventional selenium nanoparticles.

Through a series of *in vitro* and *in vivo* experiments, Wang *et al*. demonstrated that SeNExo exhibits potent therapeutic effects against TBI^[2]^. The protective effect of SeNExo injection on TBI was tested *in vivo*. SeNExo-treated TBI models showed greater improvements in spatial learning and memory in the Morris water maze test. The authors further extended the application of SeNExo to a contusive spinal cord injury (SCI) model. A significant and rapid increase in Basso Mouse Scale (BMS) scores was observed in SCI models treated with SeNExo, indicating improved locomotor performance.

On mechanism, through apolipoprotein E (APOE)-lipoprotein receptor-associated protein-1 (LRP1) interaction, SeNExo not only crosses the BBB but also accumulates and persists at the TBI site for up to 7 days, ensuring sustained antioxidant activity. The SeNExo-treated TBI group showed reduced ROS levels and neuroinflammation, with significantly fewer activated glial cells and lower levels of tumor necrosis factor alpha (TNF-α) and interleukin (IL)-1β. In the SCI model, following intravenous administration, SeNExo penetrated the blood-spinal cord barrier, accumulated at lesion sites, and was internalized by microglia, astrocytes, and neurons.

These findings establish SeNExo as a potential broad-spectrum therapeutic for CNS trauma, leveraging the synergy between exosome-mediated targeted delivery and selenium-driven antioxidative effects. *In vivo* studies using both TBI and SCI models demonstrated that SeNExo outperforms current therapeutic strategies. Compared to neural stem cell therapy or modified exosome therapy, SeNExo offers several distinct advantages. Its particles are synthesized via a one-pot approach, which holds strong potential for scalable production. Rather than modifying internal exosomal content - as most current studies focus on microRNAs (miRNAs) - SeNExo employs surface-loaded selenium nanoparticles as the active intervention. Additionally, SeNExo exhibits high BBB penetration and sustained stability at the lesion site. Together, these properties significantly enhance its potential for clinical translation.

Exosome-based interventions for TBI and SCI target key biological processes, including promoting microglial polarization toward the M2 phenotype (alternatively activated macrophages), suppressing pro-inflammatory cytokine release, and inhibiting apoptosis and oxidative damage. These therapeutic effects are primarily mediated through pathways including ROS, nuclear factor kappa-B (NF-κB), and extracellular signal-regulated kinase (ERK)^[5]^. Bioengineering on exosomes is essential to achieve these therapeutic effects and has become a rapidly emerging research hotspot in this field in recent years.

To enhance the efficacy and intervention capability of exosomes, existing studies mainly address this by modifying parent cells or directly loading specific proteins, miRNAs, small interfering RNAs, plasmids, *etc*., into exosomes using methods such as electroporation or ultrasound. In contrast, this study did not modify the exosomal cargo but instead anchored nanoselenium crystals directly onto the membrane. This creative strategy offers a novel approach, demonstrating that small inorganic particles with therapeutic potential for TBI or SCI can be functionally attached to exosomes.

As for the exosome stability and targeted delivery issues, current solutions include delivering exosomes using nanomaterials such as hydrogels, employing stem cell-derived exosomes to evade immune cell recognition, temporarily opening the BBB through techniques such as focused ultrasound, or modifying exosomes with specific peptides or antibodies. In the study by Wang *et al*., the targeting capability of SeNExo is primarily derived from the intrinsic properties of the exosomes themselves. Further modifications of SeNExo and exploration of alternative delivery routes may present important directions for improving their therapeutic efficacy^[2]^.

Given the complexity of exosomal cargo and the unpredictability of their biodistribution, safety assessments are needed to promote the clinical translation of exosome-based interventions. This study underscores several translational strengths. SeNExo retains structural integrity and biological function after lyophilization and rehydration, enabling long-term storage and “off-the-shelf” application - a major advantage for acute neurological emergencies. The use of biocompatible materials (trace-element selenium and endogenous exosomes) ensures excellent safety, as confirmed by normal hematological and histopathological findings.

Nevertheless, further investigation is necessary. For therapeutic effects, whether it arises primarily from selenium activity requires investigation. Regarding administration routes, non-invasive approaches such as intranasal delivery could improve brain targeting while minimizing systemic exposure. Although selenium was successfully loaded onto Nexo, general methods for attaching nanoparticles to exosome surfaces remain challenging and require further study. The proposed mechanism for SeNExo’s BBB penetration involves the APOE-LRP1 pathway, but it still requires in-depth experimental validation. Additionally, the interplay among SeNExo’s inorganic, proteomic, and miRNA components warrants deeper mechanistic analysis in higher animal models. Finally, it is essential for leading research institutions to establish unified safety-evaluation standards and develop guidelines to support future research and clinical translation.

In summary, this study successfully developed SeNExo, a multifunctional, cell-free nanotherapeutic prototype for CNS regeneration, by integrating neural stem cell biology with nanochemistry. Targeting efficiency and safety, however, remain major barriers to the clinical translation of exosome-based interventions for TBI and SCI. In the future, advancements in materials science and bioengineering are expected to usher in a new era of exosome-based therapeutics.

## DECLARATIONS

### AI and AI-assisted tools statement

Artificial intelligence (AI) or AI-assisted tools were used to assist with grammar revision in the preparation of this manuscript.

### Copyright

© The Author(s) 2026.
